# Baicalin chemosensitivity enhancement of cisplatin in bladder cancer via autophagy flux inhibition

**DOI:** 10.3389/fphar.2026.1676788

**Published:** 2026-03-12

**Authors:** Jiayi Zhuang, Haojie Wang, Zhaoyin Chen, Jinhua Wang, Xiaoping Zheng, Kancheng He, Dongdong Xie, Qiaoyi Chen, Shiqi Deng, Jiaqing Wu, Xiaoqing Zheng, Yingbo Dai

**Affiliations:** 1 Department of Urology, The Fifth Affiliated Hospital of Sun Yat-Sen University, Zhuhai, Guangdong, China; 2 Department of Urology, The Third Affiliated Hospital of Sun Yat-Sen University, Guangzhou, Guangdong, China; 3 Guangdong Provincial Key Laboratory of Biomedical Imaging, The Fifth Affiliated Hospital, Sun Yat-sen University, Zhuhai, Guangdong, China; 4 Department of Critical Care Medicine, The Second Affiliated Hospital of The Chinese University of HongKong/Longgang District People’s Hospital of Shenzhen, Shenzhen, Guangdong, China; 5 Department of Urology, The Second Affiliated Hospital of The Chinese University of HongKong/Longgang District People’s Hospital of Shenzhen, Shenzhen, Guangdong, China; 6 Department of Urology, The First People’s Hospital of Foshan, Foshan, Guangdong, China; 7 Department of Otorhinolaryngology, Head and Neck Surgery, The Fifth Affiliated Hospital of Sun Yat-sen University, Zhuhai, Guangdong, China; 8 Department of Kidney Transplantation, The Third Affiliated Hospital of Sun Yat-sen University, Guangzhou, Guangdong, China

**Keywords:** autophagic flux, baicalin, bladder cancer, chemosensitivity, cisplatin

## Abstract

**Background:**

Cisplatin-based chemotherapy remains the standard treatment for advanced bladder cancer (BC); however, nephrotoxicity and chemoresistance limit its clinical application. Baicalin, a flavonoid metabolite derived from the botanical drug, *Scutellaria baicalensis Georgi*, has been reported to induces autophagy in BC. However, its regulatory effect on autophagic flux under cisplatin treatment remains unclear.

**Materials and Methods:**

We used cell counting kit (CCK)-8 and 5-ethynyl-2′-deoxyuridine (EdU) assays to explore the role of baicalin in enhancing the sensitivity of BC cells *in vitro*. The underlying mechanisms were explored using transmission electron microscopy, western blotting, and immunofluorescence, and these findings were validated *in vivo.*

**Results:**

CCK-8 and EdU assays indicated that baicalin significantly enhanced the therapeutic effect of cisplatin and reduced its half maximal inhibitory concentration (IC_50_) value. Baicalin also inhibited the migration and invasion of BC cells. Western blotting indicated that baicalin suppressed cisplatin-induced autophagy by inhibiting autophagic flux, as evidenced by a reduction in the fusion of autophagosomes and lysosomes, along with decreased expression of lysosomal proteins LAMP1, LAMP2, CTSD, and CTSB. The results of the experiment *in vivo* showed that baicalin enhanced the anti-cancer effect of cisplatin. Tumor size in the combination group was significantly smaller than that in the cisplatin group.

**Conclusion:**

Baicalin enhanced the sensitivity of BC cells to cisplatin by inhibiting autophagic flux through lysosomal activity suppression. This study provides a potential botanical drug candidate for chemosensitization during BC chemotherapy.

## 1 Introduction

Bladder cancer (BC) ranks 9th among the globally prevalent cancers, with an estimated 613,791 new cases and 220,349 deaths worldwide in 2022 (Bray et al., 2024). Smoking and occupational exposure to aromatic amines and polycyclic aromatic hydrocarbons are the most significant risk factors for BC (Barone et al., 2023; Jubber et al., 2023; Barone et al., 2024). Based on the extent of muscle invasion, BC is classified as muscle-invasive bladder cancer (MIBC) or non-muscle-invasive bladder cancer (NMIBC) (Babjuk et al., 2022). Cisplatin-based adjuvant chemotherapy is the most effective strategy to improve the prognosis of patients with MIBC (Advanced Bladder Cancer Meta-analysis Collaborators, 2022). However, cisplatin is primarily eliminated through the kidneys, resulting in higher drug concentrations in the renal tissues than in other organs. This accumulation may induce both acute and chronic nephrotoxicity (Tang et al., 2023). Furthermore, due to high tumor heterogeneity, approximately half of patients with BC undergoing cisplatin-based chemotherapy experience suboptimal outcomes and may develop chemoresistance, leading to tumor recurrence or progression (Kamat et al., 2016; Alifrangis et al., 2019). Thus, enhancing cisplatin sensitivity and overcoming cisplatin resistance are essential to improve treatment efficacy. Further research is needed to explore strategies to increase cisplatin sensitivity and enable dose reduction in BC chemotherapy.

Autophagy plays a dual role in cancer development (Pérez-Hernández et al., 2019). In the early stages of tumorigenesis, tumor suppressor genes, such as p53, can effectively inhibit the abnormal proliferation of cells and thus prevent the formation of tumors by activating autophagy (Li et al., 2019; Debnath et al., 2023). The loss of tumor suppressor genes disrupts autophagy, leading to the progressive accumulation of damaged components and metabolic waste within cells. This imbalance promotes genomic instability and increases tumorigenesis risk. During the tumor treatment stage, especially when chemotherapeutic drugs cause damage to tumor cells, the tumor cells upregulate autophagy to resist the killing effect of the drugs (Pérez-Hernández et al., 2019). Currently, a large number of studies have shown that the combined use of autophagy inhibitors during the tumor chemotherapy stage can significantly enhance the therapeutic effect of chemotherapy (Chen et al., 2024).

In recent years, the extraction of anticancer plant metabolites from botanical drugs has emerged as a promising area of study, and several natural products have been shown to enhance chemotherapy sensitivity (Wang et al., 2019). Baicalin, a flavonoid derived from the dried roots of *Scutellaria baicalensis Georgi*,is widely used to treat hepatitis (Li et al., 2014), respiratory infections, and gastrointestinal diseases (Zhao et al., 2016). Increasing evidence supports the anticancer properties of baicalin via various mechanisms (Kong et al., 2021; Singh et al., 2021). Recent research has shown that baicalin kills BC cells by inducing ferroptosis (Kong et al., 2021). Simultaneously, it induces autophagy and apoptosis in T24 BC cells, thereby effectively suppressing the growth and proliferation of tumor cells (Lin et al., 2013; Hosseini, 2025). However, we failed to conduct an in-depth evaluation of the complete dynamic process of autophagic flux during baicalin-mediated tumor treatment, which limited our understanding of the role of baicalin in autophagy regulation. Although cisplatin-induced autophagy is associated with chemotherapeutic resistance in BC, the effect of baicalin on autophagic flux during cisplatin-based BC treatment has not been explored.

Given that previous studies have shown that baicalin can enhance the efficacy of chemotherapy in other cancers (e.g., non-small cell lung cancer) by regulating autophagy-related mechanisms (Chen et al., 2024), it is worthwhile to conduct an in-depth exploration of the potential of baicalin to enhance the efficacy of chemotherapy for BC.

## 2 Materials and methods

### 2.1 Cell culture

The human BC T24 and BIU-87 cells used in this study were generously provided by Prof. Fangjian Zhou of the Sun Yat-Sen University Cancer Center. Cells were cultured in RPMI-1640 medium (Gibco, United States) with 10% fetal bovine serum (Gibco, United States) and 1% penicillin/streptomycin (Procell, CHINA) at 37 °C in a 5% CO_2_ incubator. Experiments on T24 and BIU-87 cells used cells at passages 3–10 to ensure cells were in a stable proliferation state and avoid phenotypic drift caused by excessive passages.

### 2.2 Drugs and regents

Cisplatin (Selleck, S1166) was dissolved in 0.9% sodium chloride, stored at −80 °C, and diluted in the corresponding culture medium. The following drugs were dissolved in dimethyl sulfoxide (DMSO, MP, 219,605,580), stored at −80 °C, and diluted in the corresponding culture medium. The final concentration of DMSO in drug treatment was ≤0.1%. Baicalin (HPLC≥98%, Yuanye Bio-Technology, Shanghai, B20570) was applied at the concentrations of 40 μM and 80 µM for 4 h to treat T24 and BIU-87 BC cells. Prior to use, baicalin was dissolved in DMSO to prepare stock solutions, which were stored at −80 °C to avoid degradation. Chloroquine (CQ, Selleck, S6999), a lysosomotropic agent, was applied at a concentration of 25 µM for 4 h to block or diminish lysosomal acidification. The mTOR inhibitors Rapamycin (Rapa, Sigma, V900930) was applied at a concentration of 2.5 µM for 4 h to induce autophagy.

### 2.3 Cell counting Kit-8 (CCK-8) assay

CCK-8 assay was used to examine the effect of baicalin on the proliferation of BC cells and their sensitivity to cisplatin. T24 and BIU-87 cell suspensions (5,000 cells/well) were seeded in 96-well plates and treated with various concentrations of baicalin (0, 10, 20, 40, 80, 160, 240, 320 μM) and cisplatin (0, 0.1, 0.2, 0.4, 0.8, 1.6, 2.0, 2.4 μg/mL) for 24 h. CCK8 solution (10 μL) was added to each well, and the plates were incubated in an incubator (37 °C, 5% CO2) for 1 h. The absorbance was measured at 450 nm to calculate cell proliferation and determine the semi-lethal concentration of baicalin and cisplatin on T24 and BIU-87 cells, as well as the maximum non-inhibitory concentration of baicalin on T24 and BIU-87 cells. For further analysis, T24 and BIU-87 cell suspensions (5,000 cells/well) were seeded in 96-well plates and treated with baicalin (40 μM) combined with a series of concentrations of cisplatin for 24 h. CCK solution (10 μL) was added to each well, and the absorbance was measured after 1 h incubation at 37 °C.

### 2.4 Transwell assay

To investigate the effect of baicalin on cell migration, T24 and BIU-87 cells were pretreated with baicalin (40 μM) for 24 h. The cells were digested, discarded by centrifugation, and resuspended in serum-free RPMI 1640 medium. The cell density was adjusted to 5 × 10^5^/mL, and 100 μL of the cell suspension was added to the upper chamber of a transwell. RPMI-1640 medium containing serum (600 µL) was added to the lower chamber of the transwell. The cells were cultured in an incubator for 12 h, and those attached to the lower surface of the membrane were fixed with 4% paraformaldehyde for 20 min and stained with 0.1% crystal violet for 15 min. The cells were then observed in five randomly selected fields of view under a 10×light microscope, and the number of cells in each field of view was calculated and statistically analyzed using the ImageJ software. This method allowed the evaluation of the effect of baicalin on the migratory ability of BC cells.

### 2.5 Wound healing assay

T24 and BIU-87 cells in their logarithmic growth phase were digested, and approximately 2 ×10^5^ cells were added to 12 wells each. Once the cells had adhered to the bottom of the wells, a sterilized 20 μL pipette tip was used to create scratches on the cell layer along a ruler. The wells were then gently washed twice with PBS and replaced with fresh serum-free medium containing 40 μM baicalin. The 12-well plates were incubated in a conventional incubator, and photographs were taken at 0, 12, and 24 h. The migration area of the scratches was measured using ImageJ software and analyzed statistically.

### 2.6 5-Ethynyl-2′-deoxyuridine (EdU) staining assay

The effect of baicalin and cisplatin on cell proliferation was assessed using the Cell-Light TM EDU Kit (Ribobio, Guangzhou, China). T24 and BIU-87 cells were seeded into 24-well plates at a density of 3 ×10^4^ cells/well. The cells were treated with different drugs including DMSO, cisplatin (1 μg/mL), baicalin (40 μM), and cisplatin (1 μg/mL) combined with baicalin (40 μM), for 24 h. EDU staining was performed according to manufacturer’s instructions. Briefly, the steps were as follows: each well was incubated with complete medium of 1 ml containing 50 μM EDU in a cell incubator at 37 °C for 2 h, followed by fixation with anhydrous methanol for 30 min, Apollo and Hoechst staining at room temperature and protection from light for 30 min, immediately observing the image under a fluorescence microscope, and photographing 5 visual fields randomly.

### 2.7 Transmission electron microscope

T24 and BIU-87 cells were seeded in 10 cm dishes, and then treated with different drugs, including DMSO, cisplatin (1 μg/mL), baicalin (40 µM), and cisplatin (1 μg/mL) combined with baicalin (40 µM), for 24 h. The cells were scraped off using a cell scraper, and centrifuged, and the supernatant was discarded and fixed with 2.5% pentanediol at 4 °C for 3 days. The samples were sent to the Shiyanjia Laboratory (www.shiyanjia.com) for testing and analysis.

### 2.8 Immunofluorescence assay

T24 and BIU-87 cells were seeded in 12-well plates with pre-placed sterilized coverslips, and treated with different drugs (NC, Cisplatin 1 μg/mL, baicalin 40 µM and cisplatin 1 µg combined with baicalin 40 µM) for 24 h. The cells were then fixed with 4% paraformaldehyde for 30 min, 0.5%TritonX-100 (prepared in PBS) was added to the wells and allowed to permeate for 15 min at room temperature. The cells were blocked with 5% goat serum for 30 min. After that the slips were incubated with primary antibodies microtubule associated protein 1 light chain 3 Beta (LC3B) (1:1000, 83506, CST) and/or lysosome-associated membrane protein 1 (LAMP1) (1:1000, 21997, Proteintech) overnight at 4 °C. The next day, the samples were washed and incubated with Alexa Fluor 488 goat anti-rabbit IgG (1:1000, #4412, CST) or Alexa Fluor 594 anti-mouse IgG (1:1000, ab150116, Abcam) secondary fluorescent antibodies at room temperature and protected from light for 1 h. The cell nuclei were stained with DAPI (1:500) at room temperature and protected from light for 1 and 15 min. We removed the cell-climbing piece, sealed it, and observed and randomly selected five visual fields to take pictures under a confocal fluorescence microscope (Carl Zeiss, Dublin, United States); ZEN was used to process the image.

### 2.9 Western blotting

T24 and BIU-87 cells were treated with different drugs for 24 h. Cells were digested, washed with PBS, and resuspended in RIPA lysis buffer (CWBIO, China) containing 1% Protease Inhibitor Cocktail (CWBIO, China) and 1% phosphatase inhibitor cocktail (CWBIO, China) to resuspend the cells. Similarly, tumor tissues proteins extracted from tumor tissues from nude mice by adding 600 ul RIPA lysis buff which contains 1% Protease Inhibitor Cocktail (CWBIO, China) and 1% Phosphatase Inhibitor Cocktail (CWBIO, China) per 20 mg of tissue per 20 mg of tissue. The concentrations of protein samples from cells and animal tissues were determined using a bicinchoninic acid assay (Solaibao, China). Equal amounts of protein were separated by 10% sodium dodecyl sulfate-polyacrylamide gel electrophoresis (SDS-PAGE). Proteins were transferred to PVDF (Millipore, United States) and activated with methanol by semi-dry transfer (Bio-Rad, United States) under the conditions of 20 V, 0.2 A for 50 min. The membranes were blocked in 5% not-fat milk (dissolved in TBST) at room temperature for 1 h, after that incubated with the corresponding primary antibodies β-actin (1. 1000, 81115, Proteintech), lysosome-associated Membrane Protein 1 (LAMP1) (1:1000, 21997, Proteintech), Cathepsin D (CTSD) (1:1000, 55021, Proteintech), Cathepsin B (CTSB) (1:1000, 12216, Proteintech). LC3B (1:1000, 83506, CST), Phospho-SQSTM1/p62 (1:1000, 16177, CST), Lysosome-associated Membrane Protein 2 (LAMP2) (1:1000, Ab125068, Abcam) overnight at 4 °C. The primary antibodies were diluted with Western antibody diluent (P0244, Beyotime). After washing the membranes three times, they were incubated with secondary antibodies (diluted in TBST) for 1 h. The ECL kit (SQ201, Epizyme) was used to visualize the protein bands using an Invitrogen iBright FL1500 system (Thermo, United States). Finally, the resulting images were analyzed using ImageJ and Adobe Photoshop CS6 software.

### 2.10 Quantitative real-time PCR (qRT-PCR)

Total RNA was extracted from tumor cells using TRIzol (R401-01, Vazyme) according to the manufacturer’s instructions. Reverse transcription was performed with the RT Kit (RT001, ESscience), followed by quantitative real-time polymerase chain reaction using the SYBR Green Pro Taq HS Premix II qPCR Kit (AG11701, Accurate Biology). The specific primer sequences are listed as follows: LAMP1: Forward: 5′-CCG​CGG​TGT​CTT​CTT​CGT​G-3′, Reverse: 5′- TAG​AGA​CAG​CGG​CGT​TAC​CA-3’; LAMP2: Forward: 5′-GCA​GTG​CAG​ATG​AAG​ACA​AC-3′, Reverse:5′-AGTATGATGGCGCTTGAGAC-3’; CTSB: Forward: 5′-AGA​CCG​TAC​TCC​ATC​CCT​CC-3′, Reverse: 5′-CTG​TTT​GTA​GGT​CGG​GCT​GT-3'; CTSD: Forward: 5′-GTG​GAG​AGG​CAG​GTC​TTT​GG-3′, Reverse: 5′-CAC​GTT​GTT​GAC​GGA​GAT​GC-3'; LC3B: Forward: 5′-AGA​GTC​GGA​TTC​GCC​GCC​GCA-3′, Reverse: 5′- GAC​GGC​ATG​GTG​CAG​GGA​TCT; GAPDH: Forward: 5′-TGG​CAT​TGT​GGA​AGG​GCT​CA-3′, Reverse: 5′- TGG​ATG​CAG​GGA​TGA​TGT​TCT-3'.

### 2.11 Autophagosome detection by adenovirus mCherry-GFP-LC3B

T24 and BIU-87 cells were seeded in confocal dishes at a density of 30,000 cells per well. Adenovirus was added and the cells were incubated in a conventional incubator for 24 h. After that, the cells were treated with various drugs, including DMSO, baicalin (40 µM), chloroquine (25 µM), rapamycin (2.5 µM), cisplatin (1 μg/mL), and combination of cisplatin (1 μg/mL) and baicalin (40 µM) for 24 h. Next, three random visual fields were selected using a laser confocal display.

### 2.12 Lysotracker

The cells were seeded into confocal dishes at a density of 40,000 cells/well. Twenty-four hours after seeding, the cells were treated with DMSO, cisplatin (1 μg/mL), baicalin (40 μM), or a combination of cisplatin and baicalin for 24 h. Subsequently, the cells were incubated with Lysotracker™ Red DND-99 (Thermo Fisher Scientific, Cat. No. L7528) at a concentration of 50 nM for 30 min at 37 °C. Cell nuclei were stained with Hoechst stain for 30 min at room temperature, and the staining process was protected from light. Finally, five random fields of view were imaged using a confocal laser-scanning microscope (Zeiss, Model LSM880, Germany).

### 2.13 Tumor xenografts

Twenty healthy male BALB/c nude mice, aged 5 weeks and weighing 22 and 25 g, were obtained from SPF Biotechnology Co., Ltd. (Beijing, China). Subcutaneous injection of BC T24 cells (1 × 10^6^/mouse) was performed into the flanks of the mice. When the tumor volume reached 75 mm^3^ (calculated using the following formula: volume = width ^2^ × length/2), nude mice were randomly divided into four groups (n = 5) according to tumor volume using a random number table to ensure balanced grouping: control (PBS), baicalin (50 mg/kg), cisplatin (2 mg/kg), and baicalin (50 mg/kg) combined with cisplatin (2 mg/kg). Each group was subjected to identical environmental and diet conditions. Tumor volume and weight were measured by researchers unaware of the grouping information (single-blind method) to avoid measurement bias. Mice were intraperitoneally injected once every 3 days. After 21 days, the mice were weighed and euthanized. After blood collection, cervical dislocation was performed to euthanize the animals. Tumor tissues, as well as heart, liver, spleen, lung, and kidney tissues, were collected, and tumor weight and volume were recorded.

### 2.14 IHC and H&E

The tumor, heart, liver, spleen, lung, and kidney tissues were fixed in 4% paraformaldehyde (PFA), washed with PBS, embedded in paraffin, and sliced. The paraffin sections were dried in a 60 °C incubator for 1 h, dewaxed with xylene and dehydrated with gradient ethanol. EDTA Antigen Retrieval Solution (Solarbio, China, C1034) was used for antigen retrieval. Then the sections were incubated with 0.3% H_2_O_2_ for 10 min at room temperature, washed with PBS and blocked in 5% BSA at 37 °C for 30 min, followed by incubated with primary antibodies: LAMP1 (1:200, 21997, Proteintech), CTSD (1:500, ab75852, Abcam), LC3B (1:200, 83506, CST), Phospho-SQSTM1/p62 (1:200, 16177, CST) overnight at 4 °C and the secondary antibody for 1 h at room temperature. After staining with DAB (Boster, China, #SA1020) reagent for 1 min, the sections were re-stained with hematoxylin. Hematoxylin and eosin (H&E) (Solarbio, China, G1120) was performed to observe sections of the tumor tissue, heart, liver, spleen, lung, and kidney. All the images were captured using a microscope.

### 2.15 Data analysis

All experiments in this study were repeated three times, and quantitative data were expressed as mean ± standard deviation. GraphPad Prism software (version 9.0) was used for the statistical analysis. The non-paired t-test was used in the comparison between the two groups, and it was considered statistically significant when the *P* value was <0.05 (* for *P* < 0.05, ** for *P* < 0.01, *** for *P* < 0.001).

## 3 Results

### 3.1 Baicalin sensitizes cisplatin in BC cell lines

**FIGURE 1:**
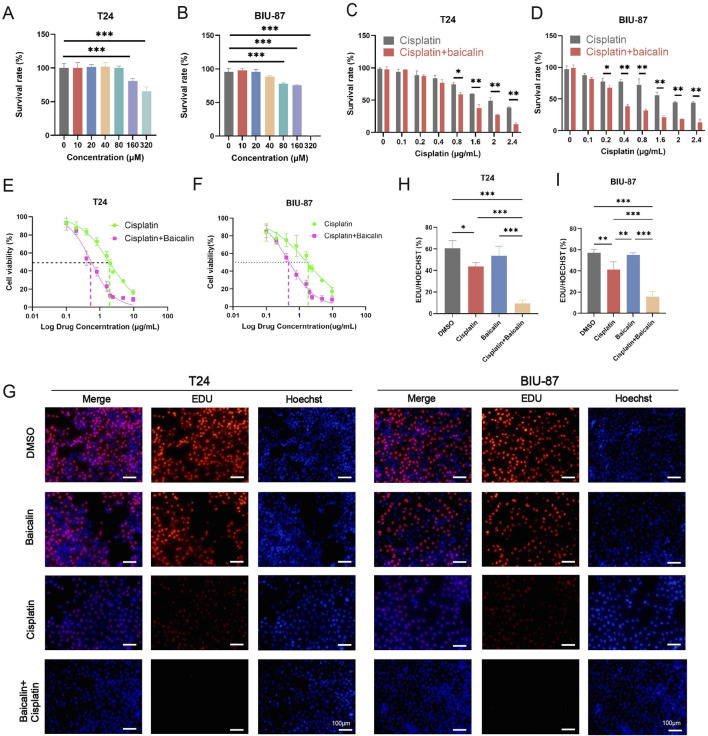
Baicalin enhances cisplatin sensitivity in human BC T24 and BIU-87 cells. **(A,B)** T24 cells and BIU-87 cells were incubated with baicalin (0, 10, 20, 40, 80, 160, 320 µM) for 24 h; The percentage of viable cells was detected by the CCK-8 assay. **(C,D)** Cotreatment of 40 µM baicalin with various concentrations of cisplatin (0, 0.1, 0.2, 0.4, 0.8, 1.6, 2, 2.4, 4.8, 9.6 μg/ml) for 24 h. The percentage of viable cells was detected by the CCK8 assay. **(E,F)** IC_50_ was calculated from the result of CCK-8 assay. **(G)** The EDU assay was performed to measure the cell viability in BC treated with baicalin (40 µM), DMSO, cisplatin (1 μg/ml), and baicalin + cisplatin for 24 h. Scale bar: 100 µm. **(H,I)** Statistical analysis of the positive rate of EDU in cell shown in Fig. D, (n = 3). All the data were presented as means ± S.D. and are representative of three independent experiments. *P*-value <0.05 was considered to be significant. *P < 0.05; **P < 0.01; ***P < 0.001.

The T24 and BIU-87 cells were treated with a series of various concentrations of baicalin (0, 10, 20, 40, 80, 160, 320 µM) for 24 h. We found no significant cytotoxic effect on BC cells at 40 µM baicalin (Figures 1A,B), which suggests that 40 µM baicalin is suitable for further studies to explore its effect on increasing cisplatin sensitivity. Human bladder T24 and BIU-87 cells were incubated with different concentrations of cisplatin (0.1, 0.2, 0.4, 0.8, 1.6, 2.4, 4.8 and 9.6 µM) for 24 h. The CCK-8 assay was performed to detect the IC_50_ of cisplatin and cisplatin combine with 40 µM baicalin in T24 and BIU-87 cells (Figures 1C,D). The IC_50_ values of cisplatin alone in T24 and BIU-87 cells were 1.616 ± 0.2889 μg/mL and 1.672 ± 0.2518 μg/mL, respectively. When combined with baicalin (40 µM), these values decreased significantly to 0.699 ± 0.1245 μg/mL (T24) and 0.5849 ± 0.1041 μg/mL (BIU-87) (*P* < 0.01, Figures 1E,F). EDU assay was performed to investigate the effect of baicalin on cisplatin sensitivity in BC cells (Figures 1H,I). The results indicated that the EDU positive rate of the combination treatment with cisplatin and baicalin was noticeably lower than that of the cisplatin group. Taken together, baicalin significantly enhanced the sensitivity of BC cells to cisplatin *in vitro.*

### 3.2 Baicalin inhibits the migration and invasion of BC

**FIGURE 2:**
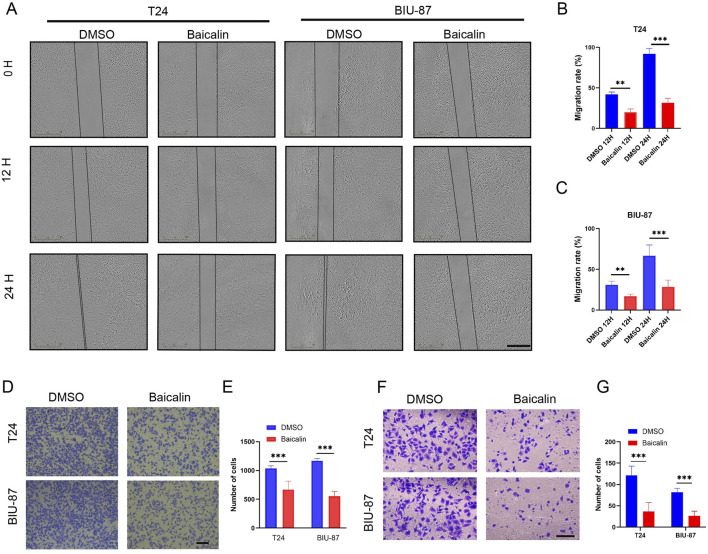
Baicalin inhibits the migration of BC cells. **(A-C)** Wound healing assays were performed to evaluate the effect of baicalin on migration ability of BC cells. The representative views of T24 and BIU-87 cells treated with DMSO or baicalin (40 µM) are shown below. Scale bar: 100 µm. **(D,E)** Transwell migration assay: Baicalin (40 µM) significantly reduced the number of T24/BIU-87 cells migrating to the lower chamber. Scale bar: 100 µm. **(F,G)** Transwell invasion assay: Baicalin (40 µM) further suppressed the invasive ability of T24/BIU-87 cells. Scale bar: 100 µm. All the data were presented as means ± S.D. and are representative of three independent experiments. **P < 0.01; ***P < 0.001.

Effects of baicalin on BC cell migration and invasion We performed wound healing and Transwell assays. The results showed that the migration rate of BC cells in the baicalin-treated group was noticeably lower than that in the control group (*P* < 0.01; Figures 2A–C). Similarly, baicalin inhibited BC cell migration to the lower chamber in the Transwell and invasion assays (*P* < 0.001, Figures 2D–H). These results indicated that baicalin inhibited the migration and invasion of BC cells *in virto*.

### 3.3 Cisplatin induces autophagy in BC

**FIGURE 3:**
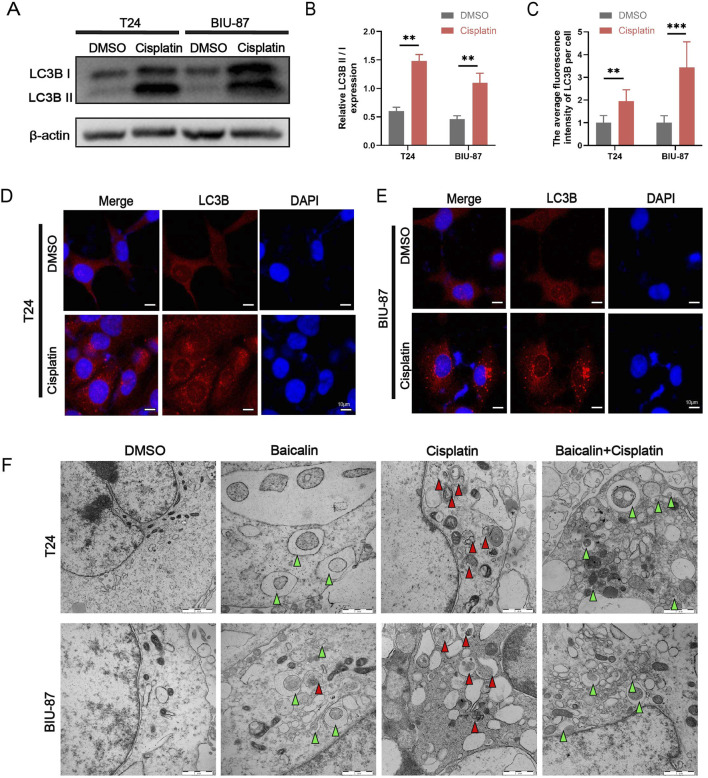
Cisplatin induces autophagy in BC T24 and BIU-87 cells. **(A,B)** T24 and BIU-87 cells were analyed by western blotting using anti-LC3B and anti-p62 antibodies after treating with cisplatin (1 μg/ml) for 24 h **(C-E)** T24 and BIU-87 cells were treated with cisplatin (1 μg/ml) for 24 h before being labeled with immunofluorescence staining of LC3B (red). The nuclei were counterstained with DAPI (blue). Scale bar: 20 µm. **(F)** The subcellular structure of BC T24 and BIU-87 cells after treatment with or without cisplatin (1 μg/ml) for 24 h were imaged by TEM (The green triangle are autophagosome, while the red triangle are autolysosomes). Scale bar: 1 μm. All the data were presented as means ± S.D. and are representative of three independent experiments. **P < 0.01; ***P < 0.001.

To validate whether cisplatin induces autophagy in BC T24 and BIU-87 cells, we measured the protein expression levels of LC3B II by western blotting (Figures 3A,B). These results suggested that the protein expression of LC3B II was significantly increased in cisplatin-treated cells (*P* < 0.01). We further performed an immunofluorescence assay to detect the fluorescence intensity of LC3B and found that the fluorescence intensity of LC3B was significantly increased in cisplatin-treated cells (*P* < 0.01, Figures 3C–E). We also performed transmission electron microscopy which indicated that a large numbers of autophagic vesicles in cisplatin-treated BC cells (Figure 3F). Autophagy is a form of cellular repair and cisplatin-induced autophagy may be associated with cellular drug resistance. Taken together, these results demonstrate that cisplatin induces autophagy in T24 and BIU-87 cells.

### 3.4 Baicalin inhibits autophagic flux in BC

**FIGURE 4:**
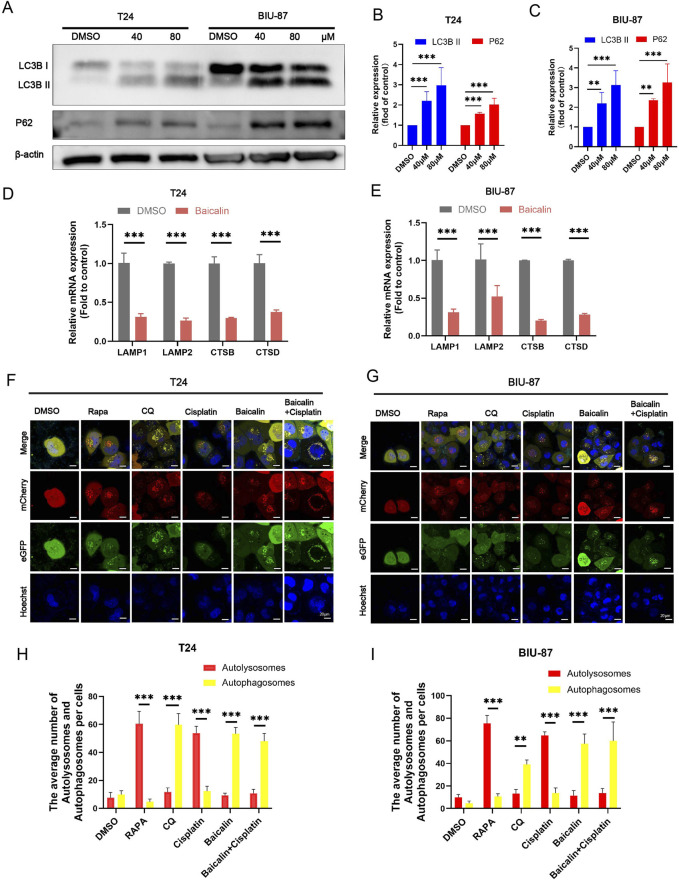
Confocal images of BC T24 and BIU-87 cells infected with adenovirus mCherry-GFP-LC3B (mCherry: red, eGFP: green, Hoechst: bule). **(A-C)** Western blot analysis of the protein level of LC3B I, LC3B II and P62 in T24 and BIU-87 cells treated with DMSO or baicalin (40 μM, 80 μM) for 24 h **(D,E)** The relative mRNA expression levels of LAMP1, LAMP2, CTSB, and CTSD were detected in T24 cells and BIU-87 cells following treatment with DMSO (as a control) or baicalin (40 μM). **(F-I)** The cells were infected with adenovirus for 24 h and then incubated with DMSO, cisplatin (1 μg/mL), Rapa (2.5 μM), CQ (25 μM), and baicalin (40 μM) for 24 h. Nuclei were stained with Hoechst. Scale bar: 20 μm. All the data were presented as means ± S.D. and are representative of three independent experiments. **P < 0.01; ***P < 0.001.

However, the effect of baicalin on autophagic flux in BC remains unclear. We examined the effect of baicalin on autophagy by detecting LC3B II protein levels in baicalin-treated cells. Treatment of T24 and BIU-87 cells increased LC3B II protein levels in a dose-dependent manner (*P* < 0.01, Figures 4A–C). In addition, western blotting revealed that the protein level of P62 was increased in baicalin-treated cells (*P* < 0.01, Figures 4A–C). P62 is a widely studied autophagic substrate. During autophagosome formation, p62 acts as a bridge linking LC3B and polyubiquitinated proteins and is selectively wrapped into the autophagosome, after which it is degraded by proteolytic enzymes in the autophagic lysosome; thus, p62 protein expression is negatively correlated with autophagic activity. Concurrently, treatment with 40 μM baicalin markedly downregulated the relative mRNA expression of LAMP1, LAMP2, CTSB, and CTSD in both T24 and BIU-87 cells (*P* < 0.01, Figures 4D,E). In addition, transmission electron microscopy indicated that autophagosomes increased and autolysosomes were largely absent in baicalin-treated cells (Figure 3F). We used the adenovirus mCherry-eGFP-LC3B system to detect the effect of baicalin on autophagic flux in BC (*P* < 0.01, Figures 4F–I). eGFP fluorescence (green) is quenched by the acidic environment in autolysosomes, while mCherry fluorescence (red) is unaffected; thus, in the mCherry-eGFP-LC3B system, the autolysosomes are red and the autophagosomes are green. In this study, CQ, a classical inhibitor of autophagy, was used to block the fusion of autophagosomes with lysosomes, and RAPA, which can induce autophagy by inhibiting the mTOR pathway (CQ was used as a positive control and RAPA was used as a negative control). These results indicated that mCherry-eGFP-LC3B is useful for detecting autophagic flux. When the cells were treated with cisplatin, the number of red puncta significantly increased. However, when the cells were treated with cisplatin and baicalin, fewer red and more yellow puncta were observed. These results indicated that baicalin blocked autophagic flux in BC cells.

### 3.5 Baicalin can inhibit lysosomal activity

**FIGURE 5:**
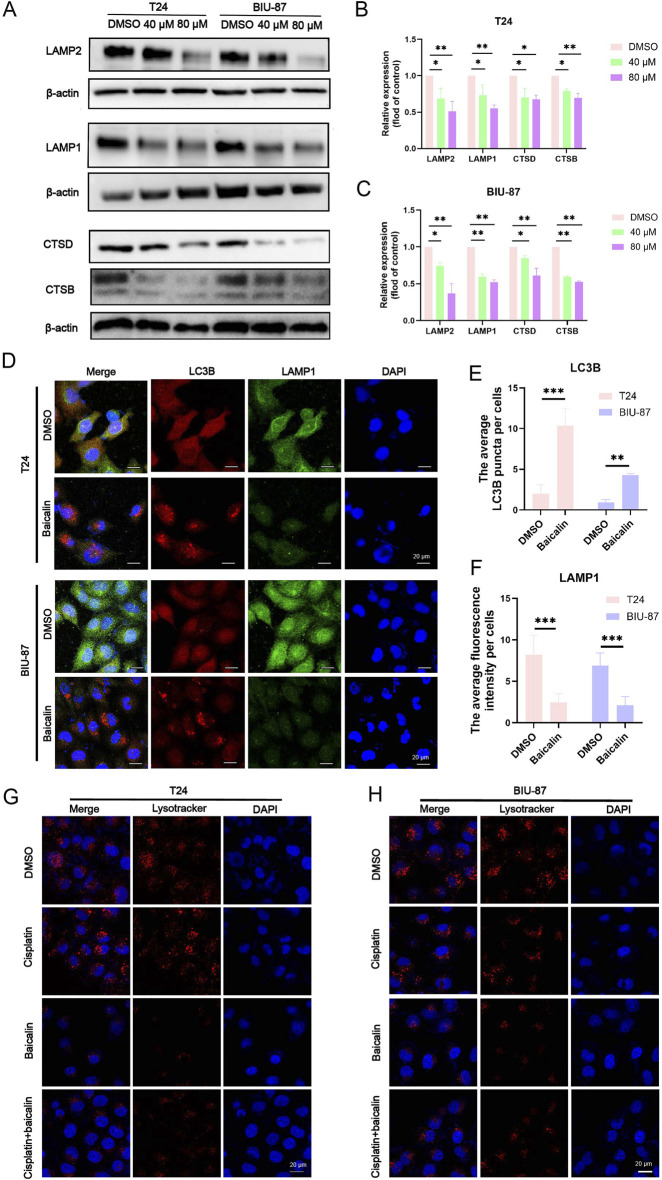
Baicalin can inhibit the binding of autophagic vesicles to lysosomes. **(A-C)** Western blot analysis of the protein level of LAMP1, LAMP2, CTSB, and CTSD in T24 and BIU-87 cells treated with DMSO or baicalin (40 μM, 80 μM) for 24 h **(D-F)** T24 and BIU-87 cells were treated with baicalin (40 μM) for 24 h. All cells were incubated with LC3B or LAMP1 antibodies before the colocalization (Red, LC3B; Green, LAMP1; Blue, Dapi). Scale bar: 20 μm. **(G,H)** T24 and BIU-87 cells were treated with DMSO, cisplatin (1 μg/mL), baicalin (40 μM), or cisplatin + baicalin for 24 h, and then stained with lysotracker (Red) for lysosomal detection, while the nuclei were stained with Hoechst (Blue). Scale bar = 20 μm. All the data were presented as means ± S.D. and are representative of three independent experiments. *P < 0.05; **P < 0.01; ***P < 0.001.

LAMP1 and LAMP2 are the main membrane proteins on the surface of lysosomes and are involved in lysosome movement. Western blotting (*P* < 0.05, Figures 5A–C), we discovered that baicalin treatment dramatically reduced LAMP1 and LAMP2 protein levels in BC T24 and BIU-87 cells. We further performed a colocalization experiment between LAMP1 and LC3B in baicalin-treated BC cells. The results indicated that baicalin caused a decrease in LAMP1 puncta and a decrease in the colocalization of LAMP1 puncta with LC3B puncta (*P* < 0.01, Figures 5D–F). To further investigate the relationship between autophagic flux blockade and lysosomal activity, we measured the protein levels of CTSB and CTSD, the main cathepsins involved in autophagic substrate degradation. CTSB and CTSD protein levels were downregulated in baicalin-treated cells. Furthermore, the use of LysoTracker, which indicates intracellular acidic compartments, revealed a significant reduction in acidic compartments in BC cells following baicalin (Figures 5G,H). These results revealed that baicalin blocked autophagic flux in BC cells by inhibiting lysosomal activity.

### 3.6 Baicalin sensitizes cisplatin in BC *in vivo*

**FIGURE 6:**
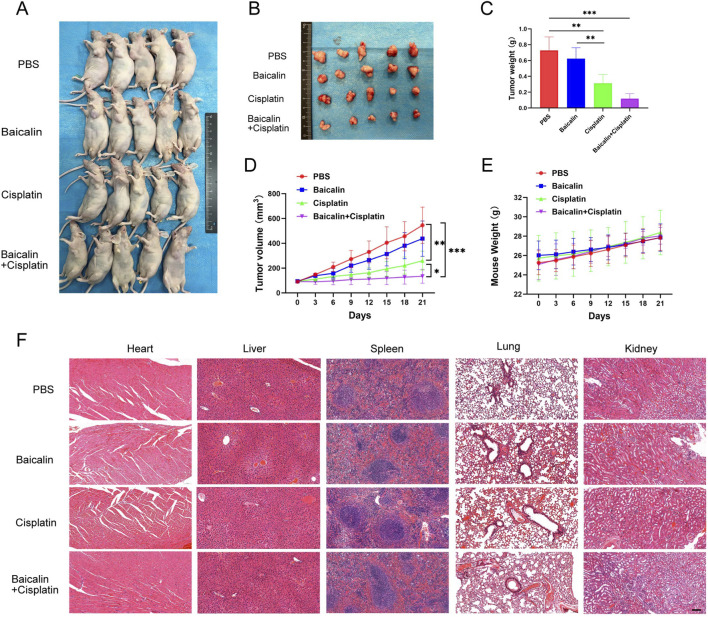
Baicalin sensitizes cisplatin in BC *in vivo*. **(A,B)** T24 cells were injected into the flank of immunodeficient mice. Tumor-bearing mice were treated with PBS, 50 mg/kg baicalin, 2 mg/kg cisplatin or cotreated with cisplatin and baicalin through intraperitoneal injection once the tumor had grown to 75 mm^3^. The tumor volume and mouse weight were measured every 3 days. After 21 days of treatment, the mice were sacrificed, and the tumors were photographed and analyzed. **(C-E)** The data of tumor volume, tumor weight, and mouse body weight were analyzed using GraphPad Prism. **(F)** Hematoxylin and eosin (H&E) staining of the heart, liver, spleen, lung, and kidney of mice treated with PBS, 50 mg/kg baicalin, 2 mg/kg cisplatin, or cotreated with cisplatin and baicalin. Scale bar: 100 μm. All the data were presented as means ± S.D. and are representative of three independent experiments. *P < 0.05; **P < 0.01; ***P < 0.001.

T24 xenograft models were established in nude mice to verify the effects of baicalin in combination with cisplatin on BC. When the tumor volume reached 75 mm^3^, the mice were randomly divided into four groups and injected with PBS, baicalin, cisplatin, or baicalin plus cisplatin. As shown in Figures 6A–D, the average tumor volume reached 545.5 ± 146.2 mm^3^ in the PBS group by the end of the experiment (day 21). In the baicalin group, the average tumor volume was 437.5 ± 142.3 mm^3^ (Figure 6D). Compared to the PBS group, tumor growth was not significantly inhibited (*P* > 0.05). In the cisplatin group, the growth of tumor volume was controlled to a certain extent, with an average volume of 251 ± 79.44 mm^3^ at the end of the experiment, showing a significant difference compared with the PBS group (*P* < 0.01). The baicalin + cisplatin group demonstrated the most remarkable inhibitory effect on tumor growth, with an average tumor volume of only 147.3 ± 78.89 mm^3^. Combined treatment with baicalin and cisplatin significantly reduced tumor volume compared to that in the PBS group (*P* < 0.001) and exerted greater antitumor efficacy than cisplatin alone (*P* < 0.05). Furthermore, we observed no significant weight loss in any of the four groups (Figure 6E), and H&E staining did not induce organic lesions in the liver, heart, spleen, lungs, or kidneys (Figure 6F). These findings suggest that baicalin enhances the sensitivity of cisplatin to chemotherapy, with few side effects *in vivo*.

### 3.7 Baicalin blocked the autophagic flux of BC *in vivo* by inhibiting lysosome activity

**FIGURE 7:**
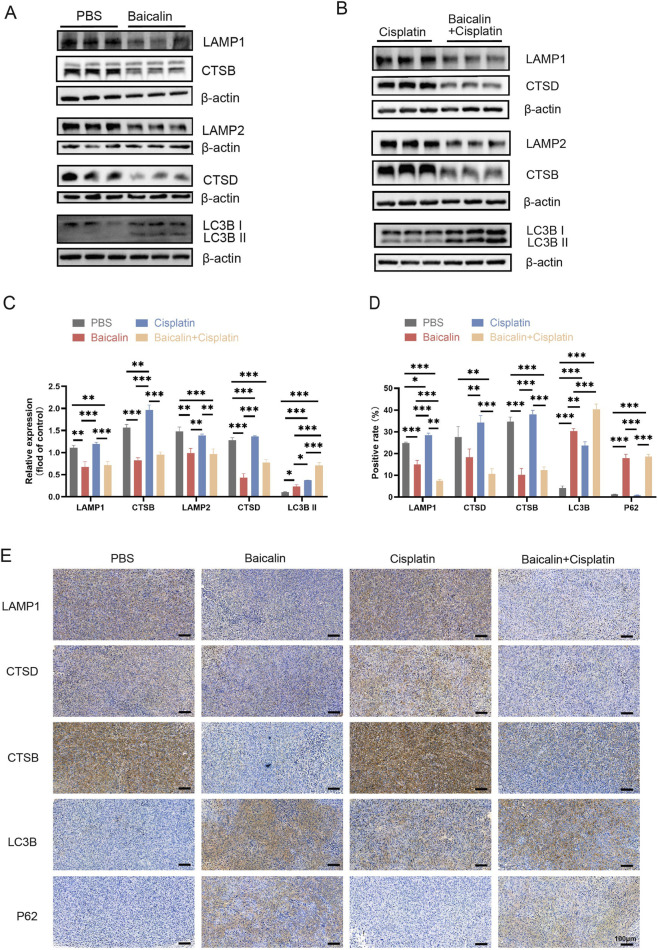
Baicalin blocked the autophagic flux of BC *in vivo* by inhibiting lysosome activity. **(A-C)** The expression of LC3B, p62, CTSB, CTSD, LAMP1, and LAMP2 of xenograft tumors was detected by western blotting. **(D,E)** Immunohistochemical staining was performed on tumor tissue using LC3B, P62, LAMP1, CTSB, and CTSD antibodies. Scale bar: 100 μm. All the data were presented as means ± S.D. and are representative of three independent experiments. *P < 0.05 vs*.* PBS; **P < 0.01 vs*.* PBS; ***P < 0.001 vs*.* PBS.

We detected lysosomes and autophagy-related proteins using western blotting (Figures 7A,B). The results indicated that the expression levels of LAMP1, LAMP2, CTSD, and CTSB were significantly decreased, but the expression levels of the autophagy-related proteins LC3B II and P62 were elevated in the baicalin-treated-and baicalin and cisplatin cotreated groups (*P* < 0.05, Figure 7C). Immunohistochemical (IHC) analysis demonstrated that compared to the control group, the tissues from baicalin- or baicalin- and cisplatin-treated mice exhibited higher expression levels of P62, and LC3B, and lower expression levels of LAMP1, CTSB, and CTSD, and cisplatin-treated mice exhibited higher expression levels of LC3B (Figures 7D,E). IHC analysis and western blotting showed that baicalin treatment decreased the expression of LAMP1, LAMP2, CTSD, and CTSB and increased the expression of LC3B and P62. These results suggested that baicalin enhanced the therapeutic effect of cisplatin *in vivo* by inhibiting lysosomal activity and blocking autophagic flux.

## 4 Discussion

Autophagy is a protective cellular mechanism that maintains homeostasis by degrading damaged organelles and misfolded proteins and recycling cellular components (Klionsky et al., 2021). It promotes cell survival under stress conditions such as starvation and hypoxia (Debnath et al., 2023). Furthermore, autophagy has been shown to have a tumor-suppressive effect, with the deletion or downregulation of autophagy-related genes leading to increased tumorigenesis and accelerated tumor progression (Yue et al., 2003; Takamura et al., 2011). However, autophagy also promotes tumor survival, drug resistance, angiogenesis, invasiveness, and metastasis (Lock et al., 2014; Kenific et al., 2016; Marsh et al., 2021; Debnath et al., 2023). This has driven efforts to inhibit autophagy as a therapeutic strategy for cancer (Wang et al., 2019). The current study sheds light on the role of autophagy in cisplatin-treated BC cells and reveals that the induction of autophagy may contribute to cancer survival.

Numerous studies have demonstrated that autophagy inhibition can effectively suppress tumor growth and enhance the efficacy of drug therapy (Goodall et al., 2014; Rebecca et al., 2017). Currently, chloroquine derivatives are the only clinically available inhibitors of autophagy that are being evaluated in clinical trials for cancer treatment (Chittaranjan et al., 2014; Pérez-Hernández et al., 2019; Ferreira et al., 2021). However, despite the evidence that many of these combinations have autophagy-inhibiting effects, HCQ failed to significantly increase progression-free and overall survival (Wolpin et al., 2014). Long-term HCQ treatment has been associated with retinopathy and cardiotoxicity. Therefore, the development of more potent and specific autophagy inhibitors is urgently required (Amaravadi et al., 2019).

Baicalin is a safe and effective drug validated in various animal models of arthritis (Wang et al., 2014), inflammatory bowel disease (Xu et al., 2021), and multiple sclerosis (Zhang et al., 2015). Baicalin inhibits BC growth by blocking the AKT/protein kinase B signaling pathway and inducing autophagic cell death (Lin et al., 2013). However, the effects of baicalin on the autophagic flux in BC remain unclear. In this study, we investigated the effect of baicalin on autophagic fluxautophagic flux using western blotting, immunofluorescence, and transmission electron microscopy (TEM). In the present study, a lower dose (40 μM) of baicalin had no significant inhibitory effect on BC, which is similar to previous studies (Lin et al., 2013). However, the combination of baicalin (40 μM) with cisplatin significantly decreased the IC50 of cisplatin in BC cells. Although previous studies have reported that baicalin induces autophagy in BC, its effect on autophagic flux has not been explored. Therefore, this study specifically examined the role of baicalin in regulating autophagic flux in BC cells.

Western blotting and immunofluorescence experiments revealed a significant increase in P62 levels in baicalin-treated BC cells. P62, a key protein in the autophagic pathway, is degraded in autophagic lysosomes when autophagic flux proceeds smoothly. Therefore, P62 serves as a marker of autophagic flux. When autophagic flux is functioning properly, P62 levels decrease and LC3B levels increase. Conversely, if autophagic flux is impaired, both P62 and LC3B levels increase (Pankiv et al., 2007; Martens, 2018; Jain et al., 2023). To further assess the effect of baicalin on autophagic flux, we used the adenovirus mCherry-GFP-LC3B, which demonstrated that baicalin impaired autophagic flux in BC cells.

Lysosomes are crucial for the later stages of autophagy, prompting us to investigate the effects of baicalin on lysosomal activity. LAMP1 and LAMP2 are the primary proteins found in the lysosomal membrane and are involved in regulating lysosomal movement, which fuses with autophagosomes to facilitate the later stages of autophagy (Eskelinen, 2006; Huynh et al., 2007). These proteins are essential for maintaining autophagic flux and their absence can disrupt the autophagic process (Eskelinen, 2006). Significantly blocked autophagic fluxautophagic flux has been observed in mouse models deficient in both LAMP1 and lAMP2 (Eskelinen et al., 2004). In the present study, baicalin decreased the protein levels of LAMP1 and LAMP2 in baicalin-treated BC cells and inhibited the fusion of autophagosomes with lysosomes. Because the regulation of autophagic flux by lysosomes is closely linked to cathepsins (Smyth et al., 2022), we explored the effect of baicalin on cathepsin activity in BC cells.

CTSD is the only cathepsin present in all human cellular lysosomes, and is essential for the degradation of autophagosomal substrates of autophagosomes (Zaidi et al., 2008; Bae et al., 2015). Western blot analysis revealed that both mature and pro-CTSD levels were reduced in baicalin-treated BC cells. CTSD plays a crucial role in maintaining autophagic flux and its downregulation can significantly impair this process (Marques et al., 2020). In addition, we examined the expression of CTSB, another cathepsin essential for substrate degradation and smooth progression of autophagic flux (Kim et al., 2022; Smyth et al., 2022). Our results indicate that the expression of mature CTSB was also reduced in baicalin-treated BC cells. Thus, baicalin may inhibit lysosomal activity and block autophagic flux by downregulating the expression of LAMP1, LAMP2, CTSD, and CTSB in BC cells.

LAMP1, LAMP2, CTSD, and CTSB are also closely related to tumor migration and metastasis in animal models and clinical studies (Mohamed and Sloane, 2006; Alessandrini et al., 2017; Mijanović et al., 2019). Accumulating evidence indicates that terminal sLeX glycans on LAMP1 and LAMP2 mediate oncogenic progression through E-selectin-dependent endothelial interactions that facilitate malignant cell adhesion, tissue infiltration, and metastatic dissemination (Tan et al., 2016). Emerging evidence has delineated the distinct proteolytic functions of cathepsin family members in oncogenic processes. Specifically, CTSD modulates glioblastoma radiosensitivity by regulating of autophagosome-lysosome fusion dynamics, while concurrently demonstrating prometastatic properties in salivary adenoid cystic carcinoma by orchestrating cytoskeletal remodeling and pseudopodia formation (Zheng et al., 2020). Parallel investigations have revealed that CTSB exerts oncogenic functions in hepatocellular carcinoma progression, enhancing malignant cell motility and invasiveness. Mechanistically, CTSB-mediated activation of the PI3K/AKT signaling axis upregulates MMP-9 expression, thereby potentiating extracellular matrix degradation and metastatic dissemination (Ruan et al., 2016). Accordingly, we performed migration and invasion assays to investigate the effects of baicalin on BC cell migration and invasion. Our findings suggest that baicalin inhibits BC cell migration. However, how these molecules (LAMP1, LAMP2, CTSD, CTSB) specifically regulate the migration and invasion of bladder cancer cells remains to be elucidated. More importantly, their critical roles require functional validation—for instance, investigating whether overexpressing LAMP1/LAMP2 or supplementing active cathepsins can reverse the baicalin-mediated chemosensitization effect.

Previous studies considered baicalin to be an autophagy inducer in BC because they only observed an increase in autophagosomes (e.g., elevated LC3B-II levels) without detecting key indicators of autophagic flux (Hosseini, 2025). Specifically, baicalin downregulated the lysosomal membrane proteins LAMP1/LAMP2 and the lysosomal proteases CTSB/CTSD (Figure 5A), thereby blocking autophagosome-lysosome fusion. Western blot analysis showed that after baicalin treatment, the levels of autophagic substrates P62 and LC3B-II were synchronously increased (Figure 4A), autophagosomes accumulated, and autolysosomes were absent (Figure 3F). The mCherry-GFP-LC3B system also demonstrated an increase in unfused autophagosomes and a decrease in autolysosomes (Figure 4F). In brief, baicalin only leads to the accumulation of autophagosomes but inhibits lysosomal activity, thereby blocking the degradation of autophagic flux. The previous conclusion that “baicalin induces autophagy in bladder cancer” and the finding of the present study that “baicalin inhibits autophagic flux” are not contradictory, but rather represent differential interpretations of the specific regulation of different stages in the autophagic process.

**FIGURE 8:**
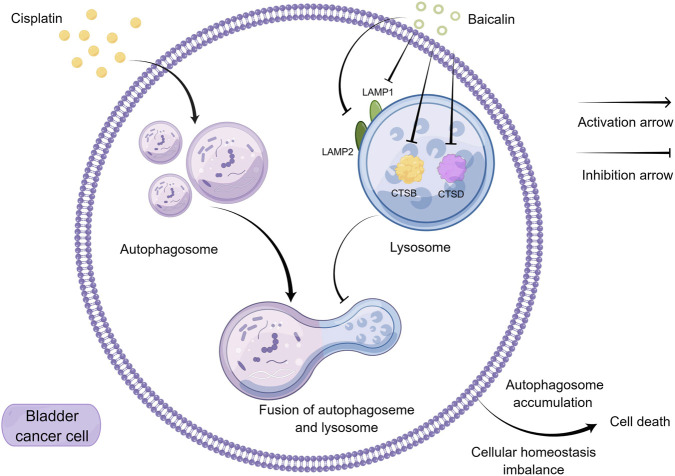
Diagram that illustrates the possible mechanism of how baicalin sensitive cisplatin in BC is shown. Cisplatin stimulates autophagy in BC. Additionally, baicalin inhibits the fusion of autophagosomes and lysosomes by inhibiting lysosomal activity. These findings suggest that baicalin could be a potential candidate for enhancing the sensitivity of cisplatin in the treatment of BC. This Figure was created by figdraw.com (ID:URTTA5b735).

Finally, we investigated the effects of baicalin on cisplatin sensitivity *in vivo* (Figure 8). The combination of baicalin and cisplatin significantly reduced the tumor size compared to the control group, as well as the cisplatin or baicalin monotherapy groups, with no observed side effects in the treated mice. Furthermore, baicalin blocked autophagic flux by downregulating the expression of LAMP1, LAMP2, CTSB, and CTSD *in vivo*. Moreover, baicalin blocked autophagic flux *in vivo* by downregulating LAMP1, LAMP2, CTSB, and CTSD. Given the side effects associated with HCQ, low-dose baicalin, a natural plant-derived compound, offers a superior safety profile. It also concurrently inhibited the migration and invasion of bladder cancer cells (Figure 2). These findings suggest that baicalin is a promising autophagy inhibitor and may serve as an effective adjuvant in cisplatin chemotherapy to sensitize bladder cancer cells. These findings suggest that baicalin is a promising inhibitor of autophagy and may serve as an effective adjuvant in cisplatin chemotherapy to enhance the sensitivity of BC cells to cisplatin.

This study has several limitations that warrant consideration. Primarily, although the animal model in this study was only constructed as a T24 cell-derived xenograft model, which has certain limitations, the response of another BC cell line (BIU-87) to baicalin was simultaneously verified in in vitro experiments: after combined treatment with baicalin, the IC_50_ of cisplatin in both cell lines decreased significantly (Figures 1E,F), and the expression trends of autophagic flux blockade-related indicators were basically consistent in the 2 cell lines (Figures 4A, 5A). The above data indicate that the regulation of the “lysosomal function-autophagic flux” axis by baicalin is not unique to T24 cells, but may cover BC subtypes with similar autophagy-dependent characteristics; however, further verification is still required to determine whether this regulatory effect is applicable to additional BC cancer cell lines with different genetic backgrounds (such as MIBC and NMIBC subtypes), and whether consistent mechanisms exist in clinical samples. Future multi-institutional clinical investigations are required to delineate appropriate patient cohorts and systematically explore synergistic combination strategies with immune checkpoint inhibitors (e.g., PD-1/PD-L1 axis modulators) in clinical settings. Although the animal experiments employed a single-dose administration regimen, which has the inherent limitation of being unable to evaluate dose-dependent effects in pharmacological research, this study serves as an early exploratory investigation that strictly adheres to the 4R ethical principles of animal experimentation and lays the foundation for subsequent in-depth research, including dose-dependent studies and multicell line validation. A notable methodological constraint is the undetermined off-target potentials of baicalin. Subsequent investigations should prioritize mass spectrometry imaging to map its spatial distribution in tumor microenvironments, complemented by genome-wide CRISPR-Cas9 screens, to systematically identify potential off-target interactions. Such mechanistic validation would substantially strengthen the pharmacological profiles of these natural plant metabolites.

## 5 Conclusion

Our results led us to conclude that baicalin enhanced the sensitivity of BC cells to cisplatin-induced cell death by disrupting autophagic flux, specifically by inhibiting the fusion of autophagosomes and lysosomes. These findings suggest that baicalin may serve as a novel adjunct therapy for bladder cancer.

## Data Availability

The original contributions presented in the study are included in the article/supplementary material, further inquiries can be directed to the corresponding authors.
